# Modulation of HIV replication in monocyte derived macrophages (MDM) by steroid hormones

**DOI:** 10.1371/journal.pone.0191916

**Published:** 2018-01-26

**Authors:** Krishnakumar Devadas, Santanu Biswas, Viswanath Ragupathy, Sherwin Lee, Andrew Dayton, Indira Hewlett

**Affiliations:** Laboratory of Molecular Virology, Division of Emerging and Transfusion Transmitted Diseases, Center for Biologics Evaluation and Research, Food and Drug Administration, Silver Spring, MD, United States of America; Universita Vita Salute San Raffaele, ITALY

## Abstract

Significant sex specific differences in the progression of HIV/AIDS have been reported. Several studies have implicated steroid hormones in regulating host factor expression and modulating HIV transmission and replication. However, the exact mechanism exerted by steroid hormones estrogen and progesterone in the regulation of HIV-1 replication is still unclear. Results from the current study indicated a dose dependent down regulation of HIV-1 replication in monocyte derived macrophages pre-treated with high concentrations of estrogen or progesterone. To elucidate the molecular mechanisms associated with the down regulation of HIV-1 replication by estrogen and progesterone we used PCR arrays to analyze the expression profile of host genes involved in antiviral responses. Several chemokines, cytokines, transcription factors, interferon stimulated genes and genes involved in type-1 interferon signaling were down regulated in cells infected with HIV-1 pre-treated with high concentrations of estrogen or progesterone compared to untreated HIV-1 infected cells or HIV-1 infected cells treated with low concentrations of estrogen or progesterone. The down regulation of CXCL9, CXCL10 and CXCL11 chemokines and IL-1β, IL-6 cytokines in response to high concentrations of estrogen and progesterone pre-treatment in HIV-1 infected cells was confirmed at the protein level by quantitating chemokine and cytokine concentrations in the culture supernatant. These results demonstrate that a potent anti-inflammatory response is mediated by pre-treatment with high concentrations of estrogen and progesterone. Thus, our study suggests a strong correlation between the down-modulation of anti-viral and pro-inflammatory responses mediated by estrogen and progesterone pre-treatment and the down regulation of HIV-1 replication. These findings may be relevant to clinical observations of sex specific differences in patient populations and point to the need for further investigation.

## Introduction

The HIV epidemic is expanding worldwide with an increasing number of distinct viral subtypes and circulating recombinant forms (CRFs). Early studies on HIV transmission were focused mainly on males, typically transmission between males who have sex with males (MSM) and majority of the outcomes and conclusions about pathogenesis, disease progression and therapeutic options were derived from studies conducted on male populations. At present, there is very little information on HIV pathogenesis, transmission and disease progression that impacts the female population. The current pattern of HIV transmission is changing, with heterosexual transmission now accounting for greater than 70% of the new cases identified worldwide, leading to a greater number infections in women. In addition, studies performed in Kenya indicate that there may be sex-based differences in HIV-1 diversity at the time of infection [1, 2]. In the US, the estimated number of AIDS cases increased 15% among women and only 1% among men from 1999 to 2003. It has also recently been reported that HIV viral load in blood is generally lower in women than in men at similar stages of HIV infection. Women appear to be more susceptible to HIV-1 infection, but have lower plasma viral RNA levels and a more diverse population of HIV-1 variants [3]. In addition to HIV-1 infection, sex based differences in susceptibility to other human viral infections (HSV-2, measles virus, Hantaviruses and vesicular stomatitis virus) have been reported [4, 5].

HIV replication is dependent on the host transcriptional machinery and many of these host factors are determinants of cell tropism and host range of HIV and could positively or negatively regulate HIV replication. Several studies have implicated steroid hormones estrogen and progesterone in influencing HIV transmission and modulating HIV replication [6–14]. Reports indicate that the use of progesterone based injectable hormonal contraceptives is associated with an immunosuppressive female genital tract environment that could influence susceptibility to HIV infection [15]. Similarly, other studies have implicated steroid hormones estrogen and progesterone in having a profound influence on immune cells leading to the modulation of anti-viral innate immune responses that impact the transmission, replication and disease progression during the course of HIV-1 infection [16–22]. Although there are several studies that associate steroid hormones and HIV-1infection, the exact mechanism exerted by estrogen and progesterone in the regulation of HIV-1 transmission and replication is still unclear. In our report, we have investigated the effects of hormones estrogen and progesterone on the replication of HIV in monocyte derived macrophages. Our study suggests a strong correlation between the down-modulation of anti-viral and pro-inflammatory responses to HIV-1 infection mediated by estrogen and progesterone pre-treatment and the down regulation of HIV-1 replication.

## Materials and methods

### Reagents

17β-estradiol, Cat # E2257; progesterone, Cat # P7556; mifepristone, Cat# M8046; and tamoxifen, Cat # T5648 were purchased from Sigma Chemical Company, St. Louis, MO, USA. Macrophage colony stimulation factor (M-CSF), Cat # PHC2044 was purchased from Thermo Fisher Scientific, Waltham, MA, USA.

### Isolation and culture of monocyte-derived macrophages (MDMs)

Human monocytes isolated from PBMC after leukophoresis of female and male donors seronegative for both HIV-1 and hepatitis B and purified by countercurrent centrifugal elutriation were provided by the NIH Blood bank. This study was approved by the NIH ethics committee (study number: 99-CC-0168, PI: Susan F. Leitman, M.D.). Written informed consent was obtained from healthy, normal blood donors for publication of this Case Report and any accompanying images according to the ethical principles of international ethical guidelines for biomedical research involving human subjects. A categorical exemption is in place for CBER / FDA for experimental studies by CBER/FDA researchers using existing, deidentified samples of blood and /or blood products originally obtained under the NIH IRB-approved protocol and consent form 99-CC-0168. The cells were cultured for 5 days in DMEM supplemented with 10% FBS, 100 units/ml Penicillin and 100 μg/ml Streptomycin/ml and 0.02 μg/ml macrophage colony stimulation factor (M-CSF) as described earlier [23]. After 5 days the culture media were removed and replaced with complete media containing 10% charcoal stripped FBS and cultured for an additional 24 hours. After 24 hours appropriate concentrations of estrogen or progesterone were added to the culture media and the cells were cultured for an additional 5 days before infection with HIV-1.

### Infection with HIV-1

Primary monocyte-derived macrophages (MDMs) isolated from donors and cultured for 5 days were infected with aliquots of HIV-1 Ba-L and primary isolates HIV-1 subtype B-NSI (B9697) and HIV-1 subtype C-NSI (SE/364/90) equivalent to 5 ng/ml p24 units [10]. After a two-hour exposure, virus particles were removed and the cells were washed 3 times in 1x PBS. Fresh culture medium was added with appropriate additions of the steroid hormones and cells cultured at 37° C until further use. The following concentrations of steroid hormones were used: Estrogen: 1.75 μM, 110 nM, 140 pM, 40 pM; Progesterone: 64 nM, 32 nM, 2.5 nM, and 1 pM. Culture supernatants were collected at specified days post infection (day 9 and day 12) and HIV-1 replication quantitated by p24 ELISA. After supernatants were harvested, an equivalent volume of growth media containing appropriate concentrations of steroid hormones was added to each well to maintain the effective concentration of steroid hormones throughout the experiment.

### Quantitation of HIV-1 replication

Culture supernatants were assayed for HIV-p24 using a NEN/DuPont ELISA analysis kit (Perkin Elmer Life Sciences, Inc., Boston, MA, USA) according to the manufacturer’s instructions. Assays were performed in triplicate.

### Treatment with estrogen and progesterone antagonists

MDMs were pre-treated with 0.6 nM Mifepristone or 5 μM Tamoxifan for 2 hours before adding 110 nM estrogen or 64 nM progesterone to the culture media. The MDMs were cultured for 5 additional days in the presence of antagonists and hormones and infected with HIV-1 Ba-L. Culture supernatants were collected 9 days post infection and HIV-1 replication quantitated by p24 ELISA.

### RNA isolation and real-time quantitative PCR array

Total RNA was extracted from samples using the RNeasy plus mini kit (Qiagen, Germantown, MD, USA). Three hundred nanograms of total RNA from each sample was reverse transcribed into cDNA using the RT^2^ first strand kit (Qiagen, Germantown, MD, USA). An RT^2^ Profiler PCR Array (Cat#122z Qiagen, Germantown, MD, USA) was used to examine the mRNA levels of different antiviral response genes. House-keeping genes β-actin, glyceraldehyde 3-phosphate dehydrogenase, β₂ macroglobulin, hypoxanthine phosphoribosyl transferase 1 and ribosomal protein lateral stalk subunit P0 used for normalization. Several negative controls were included in each run. All PCR experiments were conducted with a ViiA 7 Real Time PCR system (Thermo Fisher Scientific, Waltham, MA, USA). Array plates included endogenous controls, reverse transcriptase negative controls, and genomic DNA contamination controls. Ct values of 35 or greater for any particular mRNA in either the control or the experimental samples were excluded from the analysis and marked as undetectable or undetermined. The Ct values that met these stringent criteria were uploaded into the Qiagen/SABiosciences software (RT^2^ Profiler PCR Array Data Analysis) and the fold regulation was calculated for each mRNA. Data analysis was performed using the ΔΔCt based calculations. Assays were performed with RNA samples isolated from MDMs obtained from 3 independent donors.

### Bioplex cytokine and chemokine Assay

BioPlex Pro Human Cytokine and Chemokine multiplex assay was used to quantitate IFN-α2, IFN-β, IFN-γ, CXCL8, CXCL9, CXCL10, CXCL11, CCL3, RANTES, TNF-α, IL-1β, IL-6, IL-12β and IL-18 (Bio-Rad Laboratories, Hercules, CA, USA) using MagPlex beads was performed using frozen cell culture supernatants in a flat bottom 96-well plate according to the manufacturer’s instructions. Briefly, 50 μL of coupled magnetic bead mixture was added to each well and washed with wash buffer. All wash steps were performed using BioPlex Pro™ Wash Station (Bio-Rad Laboratories, Hercules, CA, USA). 50 μL of undiluted samples and appropriate concentrations of standards were prepared and added in duplicate to the beads. The 96-well plate was incubated on a shaker for 2 hours at 850 rpm at room temperature. After incubation, the plate was washed three times with 100 μL of wash buffer using the wash station. Detection antibodies were added to each well and the plate incubated on a shaker at 850 rpm for 30 minutes at room temperature. The plate was washed once again and streptavidin-phycoerythin solution added to the wells. Beads were resuspended in assay buffer and incubated for 10 minutes at room temperature. The signal was captured using a Luminex 200 instrument (Luminex Corporation, Austin, TX, USA) and results were analyzed using the BioPlex Pro software (Bio-Rad Laboratories, Hercules, CA, USA). A standard curve was used to determine the absolute concentration of each analyte. Points on the standard curve with recovery below 70% or above 130% were considered invalid. Curve fit was made with a weighted 5PL method.

### Ingenuity pathway analysis

To further understand the function between hormone pre-treatment and HIV-1modulation, we used Ingenuity Pathway Analysis (IPA) comparison network tools to demonstrate their differences. Data sets were analyzed through the use of QIAGEN's Ingenuity® Pathway Analysis (IPA®, QIAGEN Redwood City, CA, USA www.qiagen.com/ingenuity). Identification of the canonical pathways most significantly associated with the genes differentially expressed between MDMs that were both pre-treated with estrogen or progesterone and infected with HIV-1 and MDMs infected with HIV-1 not treated with estrogen or progesterone was done using Ingenuity Pathways Knowledge Base (IPKB). The differentially expressed genes identified by the PCR array were used for the analysis. The significance of association was measured on the basis of the ratio of the number of genes from the data set that map to the pathway divided by total number of genes that map to the canonical pathway (as displayed); and a P-value determining the probability that the association between the genes in the data set and the canonical pathway is explained by chance alone (Fischer’s exact test).

Upstream Regulator Analysis was used to identify upstream regulators and predict whether the upstream regulators were activated or inhibited in the experimental data set relative to the control. An overlap p-value is computed based on significant overlap between genes in the dataset and known targets regulated by the transcriptional regulator. The significance of the association is measured using a z-score algorithm designed to reduce the chance that random data will generate significant predictions. Z-score is a statistical measure of correlation between relationship and gene expression. A z-score > 2 or < -2 is considered significant. Diseases and Functions analysis (downstream effects) was used to identify whether cellular processes and biological functions are upregulated or down regulated in the experimental data set. The predicted directional changes in cellular processes and biological functions are determined by correlating the gene expression pattern in the experimental data set with experimental gene effects reported in the literature.

## Results

**Fig 1:**
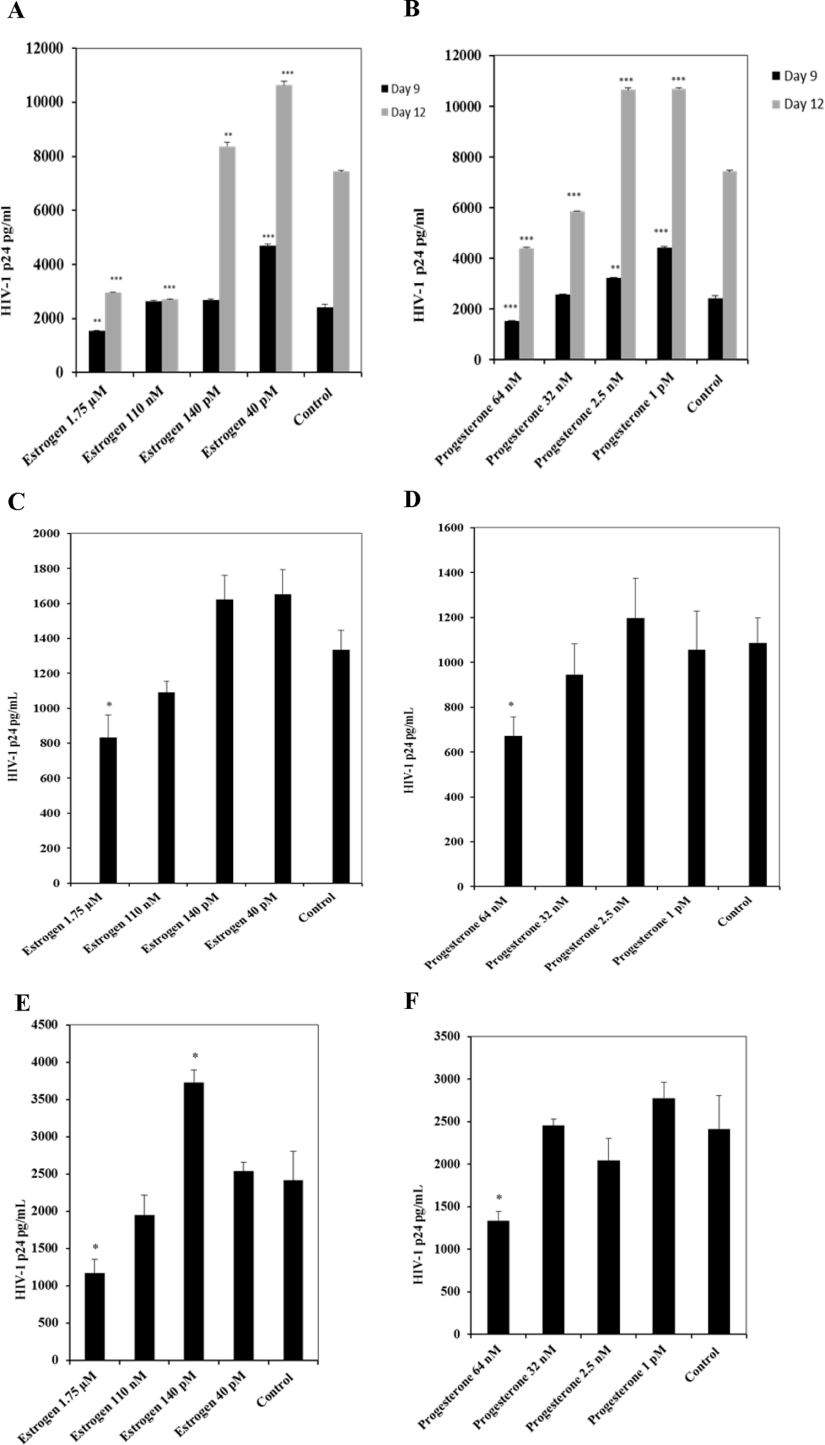
Modulation of HIV-1 replication by estrogen and progesterone. MDMs (1x10 ^6^ cells/well) were pre-treated with appropriate concentrations of estrogen or progesterone and infected with HIV-1. After 2 hours the virus was removed, and fresh culture media added with appropriate hormones and cultured. Hormone concentrations used: Estrogen: 1.75 μM, 110 nM, 140 pM, 40 pM; Progesterone: 64 nM, 32 nM, 2.5 nM and 1 pM. Culture supernatants were collected 9 days and / or 12 days post infection and HIV-1 replication quantitated by p24 ELISA. Culture supernatants were analyzed in triplicate. Results expressed as mean ± SEM are representative of three independent experiments. Asterisk (*) over the bars indicates significant difference with control, *** P <0.0001, ** P <0.001 and * P <0.05. P-values were generated by Student’s t-test and comparisons were done with control. A. MDMs infected with HIV-1 BaL at 5 ng/ml and pre-treated with estrogen. B. MDMs infected with HIV-1 BaL at 5 ng/ml and pre-treated with progesterone. C. MDMs infected with HIV-1 Clade B NSI at 5 ng/ml and pretreated with estrogen. D. MDMs infected with HIV-1 Clade B NSI at 5 ng/ml and pre-treated with progesterone. E. MDMs infected with HIV-1 Clade C NSI at 5 ng/ml and pretreated with estrogen. F. MDMs infected with HIV-1 Clade C NSI at 5 ng/ml and pre-treated with progesterone.

In vitro experiments carried out with MDMs isolated from donors that were treated with different concentrations of steroid hormones estrogen (1.75 μM, 110 nM, 140 pM, and 40 pM) and progesterone (64 nM, 32 nM, 2.5 nM, and 1 pM) infected with HIV-1 BaL and primary isolates representing different HIV-1 subtypes B and C, demonstrated modulation of HIV-1 replication. High concentrations of estrogen and progesterone down regulated HIV-1 replication (Fig 1A–1F). Greater than 2 fold decrease (a 60% decrease) in HIV-1 replication was observed in MDMs infected with HIV-1 BaL pre-treated with 1.75 μM estrogen and 45% down regulation of HIV-1 replication was observed in MDMs infected with HIV-1 BaL pre-treated with 64 nM Progesterone (Fig 1A and 1B). Consistent with reports in literature [24], a slight increase in HIV-1BaL replication was observed in MDMs pre-treated with low concentrations of estrogen (140 pM and 40 pM) and progesterone (2.5 nM and 1 pM). A similar trend was observed with MDMs pre-treated with estrogen or progesterone and infected with HIV-1 subtype B and HIV-1 subtype C (Fig 1C, 1D, 1E and 1F).

**Fig 2:**
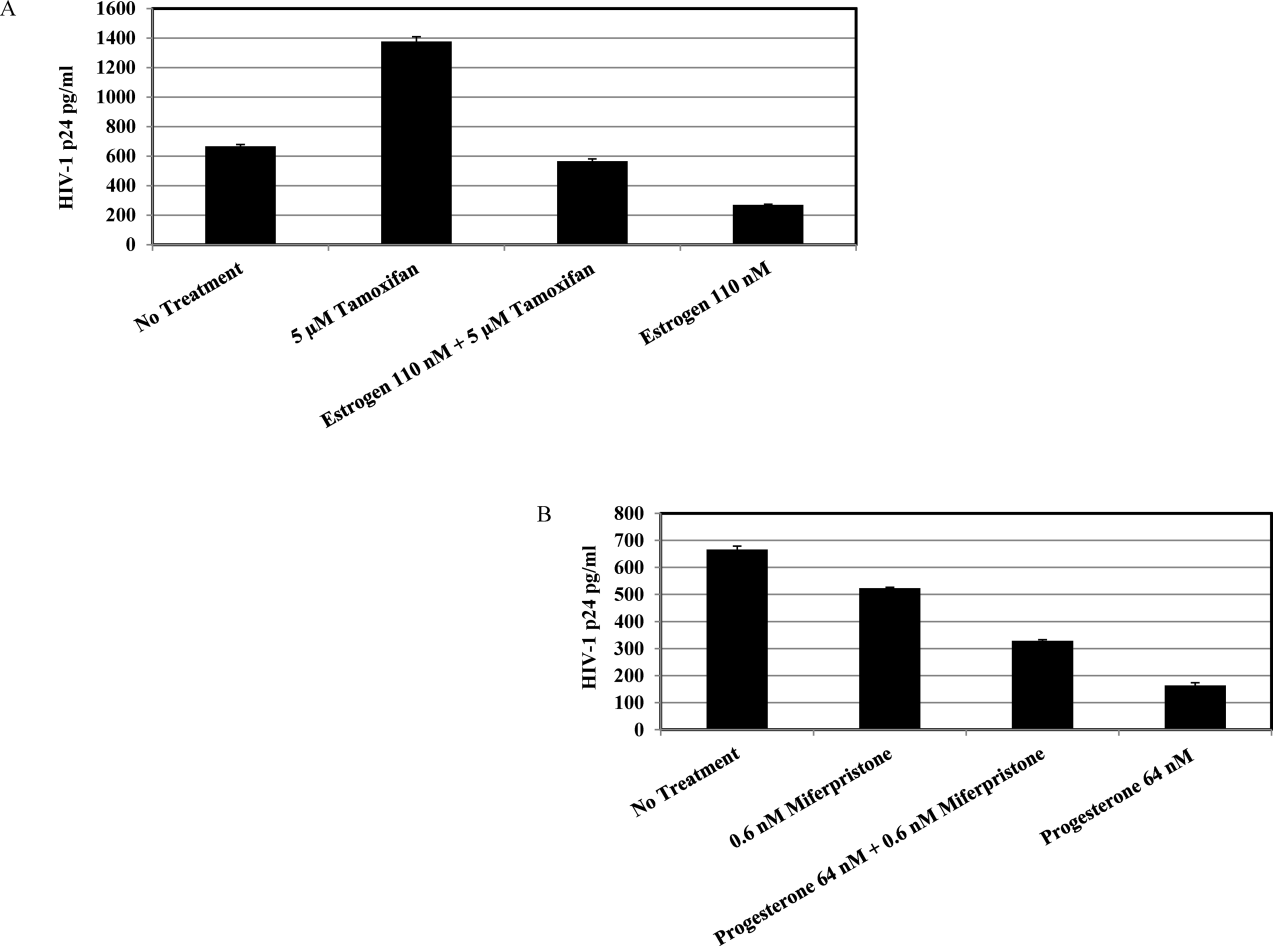
HIV-1 BaL replication in MDMs treated with mifepristone and tamoxifen. MDMs (1 x10 ^6^ cells/well) were pre-treated with concentrations of 5 μM tamoxifen or 0.6 nM mifepristone for 2 hours and appropriate concentrations of estrogen or progesterone were added and cultured for 5 days. The cells were infected with HIV-1 BaL at 5 ng/ml and cultured in media containing 110 nM estrogen and 64 nM progesterone. Culture supernatants were harvested 9 days post infection and HIV-1 replication quantitated by p24 ELISA. Culture supernatants were analyzed in triplicate. Results expressed as mean ± SEM are representative of three independent experiments.A. Tamoxifen and estrogen pre-treated MDMs B. Mifepristone and progesterone pre-treated MDMs.

To further study the mechanisms involved in the down regulation of HIV-1 replication by estrogen and progesterone, MDMs were treated with mifepristone, a steroidal anti progestogen, which acts as a competitive progesterone receptor antagonist and tamoxifen, an estrogen receptor antagonist prior to treatment with high concentrations of estrogen and progesterone. Results indicated that pre-treatment with mifepristone and tamoxifen reversed the inhibitory effects mediated by estrogen and progesterone treatment (Fig 2A and 2B). Thus, consistent with other reports in literature [24], data implies that the inhibitory effect of estrogen and progesterone maybe mediated through the engagement of estrogen and progesterone receptors. However, this does not exclude the possibility of involvement of other receptors like G protein-coupled receptors, ion channels and SHBG/SHBG-R complex [25–28].A probable mechanism for the regulatory effect exerted by estrogen and progesterone could be due to modulation of chemokine receptors that serve as co-receptors for HIV-1 entry [29–31]. To determine the effects of estrogen and progesterone on down regulation of CD4 and CCR5, MDMs were treated with high concentrations and low concentrations of estrogen and progesterone and stained with anti-CD4, anti-CCR5 antibodies and subjected to FACs analysis. The results (S1 Fig) indicated that there was no detectable up or down regulation of CD4 or CCR5 receptors at the concentrations of the steroid hormones tested.

**Fig 3:**
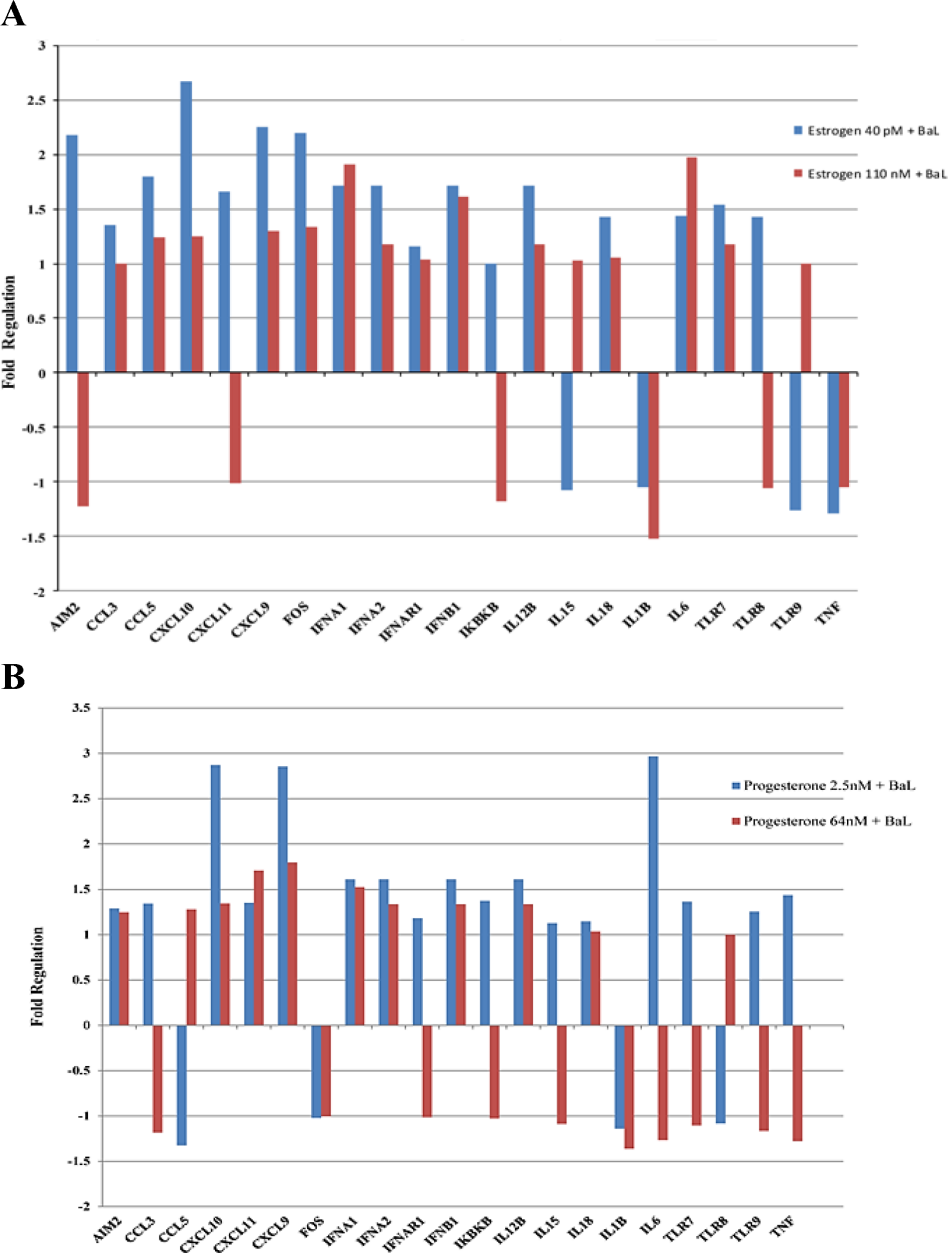
Identification of differentially expressed genes in estrogen or progesterone treated MDMs infected with HIV-1 using RT^2^ Profiler PCR Arrays. Assays were performed with experimental RNA samples isolated from MDMs obtained from 3 independent donors. A. Differentially expressed genes in estrogen treated MDMs infected with HIV-1 BaL.B. Differentially expressed genes in progesterone treated cells.

To further elucidate the molecular mechanisms associated with the down-regulation of HIV-1 replication by estrogen and progesterone, gene expression profiling of genes involved in innate antiviral immune response such as toll-like receptors, nucleotide-binding oligomerization domain (Nod)-like receptors, retinoic acid-inducible gene 1 (IRIG-1)-like receptors, receptor signaling effectors, genes involved in type-1 interferon signaling, interferon stimulated genes (ISG) including inflammatory cytokines, major chemokines, transcription factors and other host factors in response to HIV-1 infection were analyzed using PCR arrays. The results indicate that several chemokines, cytokines, transcription factors, interferon stimulated genes (ISG) and genes involved in type-1 interferon signaling were found to be up regulated in HIV-1 infected MDMs not treated with estrogen or progesterone (S2 Fig). Many of these genes were found to be differentially expressed in MDMs infected with HIV-1 pre-treated with estrogen or progesterone compared to HIV-1 infected MDMs not treated with estrogen or progesterone (Fig 3A and 3B). Results presented in Fig 3A and 3B, indicate that in HIV-1 infected cells treated with high concentrations of estrogen (110 nM) or progesterone (64 nM) certain inflammatory chemokines, cytokines and host response genes were down regulated compared to cells treated with low concentrations of estrogen (40 pM) or progesterone (2.5 nM). Notably, cytokines IL-1β and IL 18, chemokines CXCL9, CXCL10 and CXCL11, and other host response genes like AIM2 and FOS were down regulated in HIV-1 infected cells treated with high concentration of estrogen (110 nM). In contrast, HIV-1 infected cells treated with low concentration of estrogen (40 pM) exhibited up regulation of these cytokines, chemokines and host response genes compared to HIV infected cells without estrogen treatment. Similarly, treatment with high concentration of progesterone (64 nM) resulted in down regulation of inflammatory chemokines, cytokines and host response genes. Specifically, cytokines IL-18, IL-6, IL-1β, TNF-α, and Interferon responsive genes CXCL9 and CXCL10 were down regulated only in HIV-1 infected cells treated with high concentration of progesterone. No changes in gene expression were identified (data not shown) in uninfected cells treated with estrogen or progesterone, implying that estrogen or progesterone treatment modulates the secretion of host inflammatory cytokines and chemokines produced in response to HIV-1 infection.

**Fig 4:**
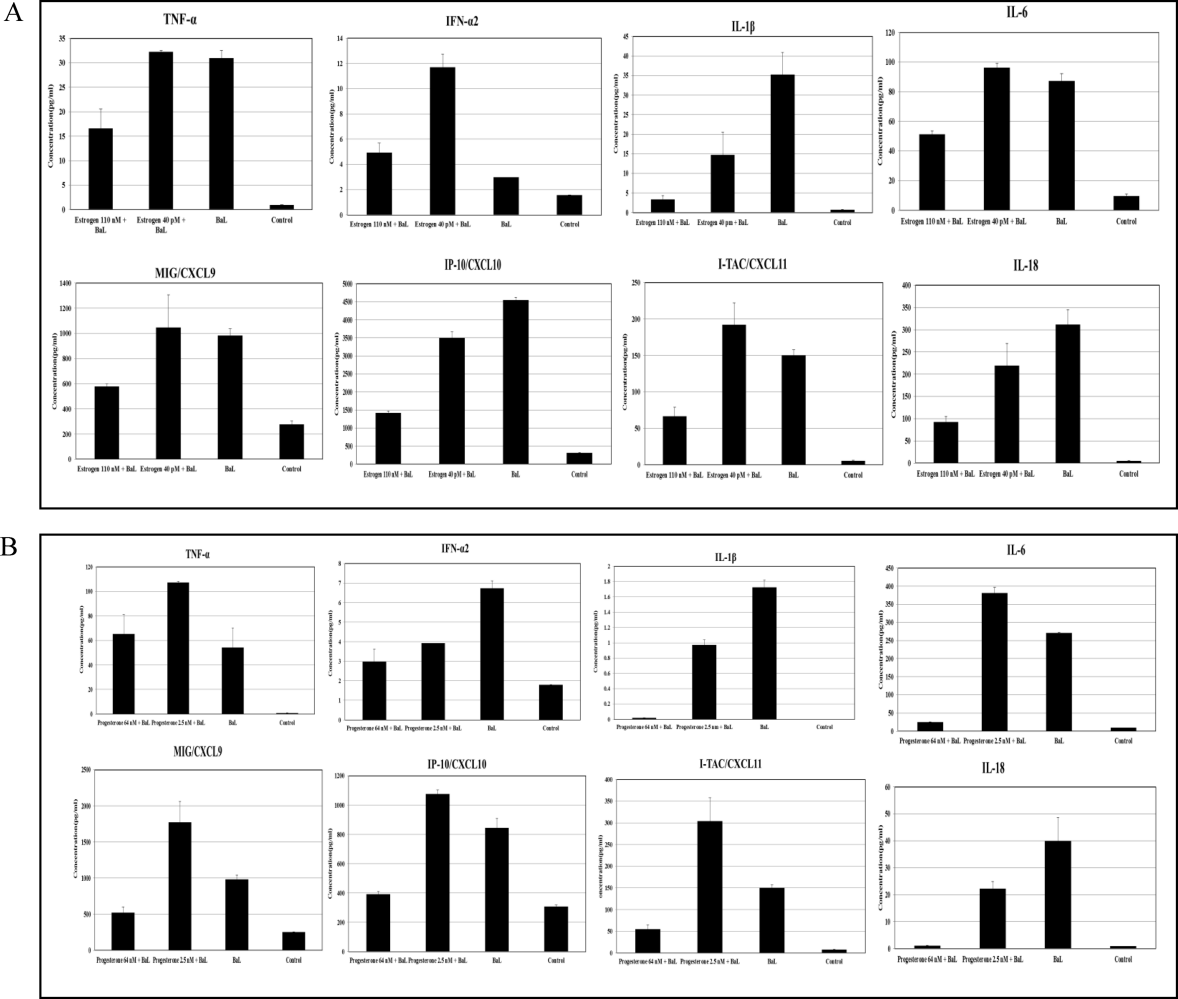
BioPlex analysis of secreted cytokines and chemokines in culture supernatants. **A.** BioPlex analysis of secreted cytokines and chemokines in culture supernatants from monocytes derived macrophage cells infected with HIV-1 (BaL) pre-treated with 40 pM or 110 nM estrogen and MDMs not infected with HIV-1, not pre-treated with estrogen as control. Presented in this panel are Tumor necrosis factor-α (TNF-α), Interferon-α2 (IFN-α2), Chemokine (C-X-C motif) ligand 9 (CXCL9), Chemokine (C-X-C motif) ligand 10 (CXCL10), Chemokine (C-X-C motif) ligand 11 (CXCL11), Interleukin 1 β (IL1β), Interleukin-6 (IL-6), Interleukin-18 (IL-18). Culture supernatants were analyzed in duplicate. Results expressed as mean ± SEM are representative of three independent experiments. **B.** BioPlex analysis of secreted cytokines and chemokines in culture supernatants from monocytes derived macrophage cells infected with HIV-1 (BaL) pre-treated with 2.5 nM or 64 nM progesterone and MDMs not infected with HIV-1, not pre-treated with progesterone as control. Presented in this panel are Tumor necrosis factor-α (TNF-α), Interferon-α2 (IFN-α2), Chemokine (C-X-C motif) ligand 9 (CXCL9), Chemokine (C-X-C motif) ligand 10 (CXCL10), Chemokine (C-X-C motif) ligand 11 (CXCL11), Interleukin 1 β (IL1β), Interleukin-6 (IL-6), Interleukin-18 (IL-18). Culture supernatants were analyzed in duplicate. Results expressed as mean ± SEM are representative of three independent experiments.

To validate the differentially expressed cytokine and chemokine genes in response to estrogen and progesterone treatment identified by the PCR array we quantitated the expression levels of interferons (IFN-α2 and IFN-γ), chemokines (CXCL8, CXCL9, CXCL10 and CXCL11) and cytokines (CCL3, IL-1β, IL-6, IL-12B, IL-18, RANTES and TNF-α) at the protein level, in culture supernatants from HIV-1 infected cells treated with 110 nM estrogen (high concentration) and 40 pM estrogen (low concentration) or 64 nM progesterone (high concentration) and 2.5 nM progesterone (low concentration). Our results (Fig 4A) indicate that the levels of cytokines IL-1β, IL-6, IL-18 and TNF-α; and chemokines CXCL9, CXCL10 and CXCL11 were down regulated in culture supernatants collected from HIV-1 infected MDMs treated with high concentrations of estrogen (110 nM) compared to untreated HIV-1 infected cells or HIV-1 infected cells treated with low concentrations of estrogen (40 pM). Similarly, results (Fig 4B) indicate that the levels of cytokines IL-1β, IL-6, IL-18 and chemokines CXCL9, CXCL10 and CXCL11 were down regulated in culture supernatants collected from HIV-1 infected MDMs treated with high concentrations of progesterone (64 nM) compared to untreated HIV-1 infected cells. Pre-treatment with 64nM progesterone down regulated TNFα levels compared to HIV-1 infected MDMs pre-treated with 2.5 nM progesterone. Similarly, pre-treatment with low concentrations of estrogen was associated with a slight increase in the secretion of chemokines CXCL9 and CXCL11 and pro-inflammatory cytokines IL-6 and TNF-α (Fig 4A) and pre-treatment with low concentrations of progesterone increased the secretion of chemokines CXCL9, CXCL10/IP-10 and CXCL11 and pro-inflammatory cytokines IL-6 and TNF-α (Fig 4B). Pre-treatment with 64 nM and 2.5 nM progesterone decreased the levels of IFN-α2 (Fig 4B) compared to untreated HIV-1 infected MDMs. No significant changes were found in the levels of CCL3, IL-12β, RANTES and CXCL8 in response to estrogen or progesterone treatment (data not shown).

**Fig 5:**
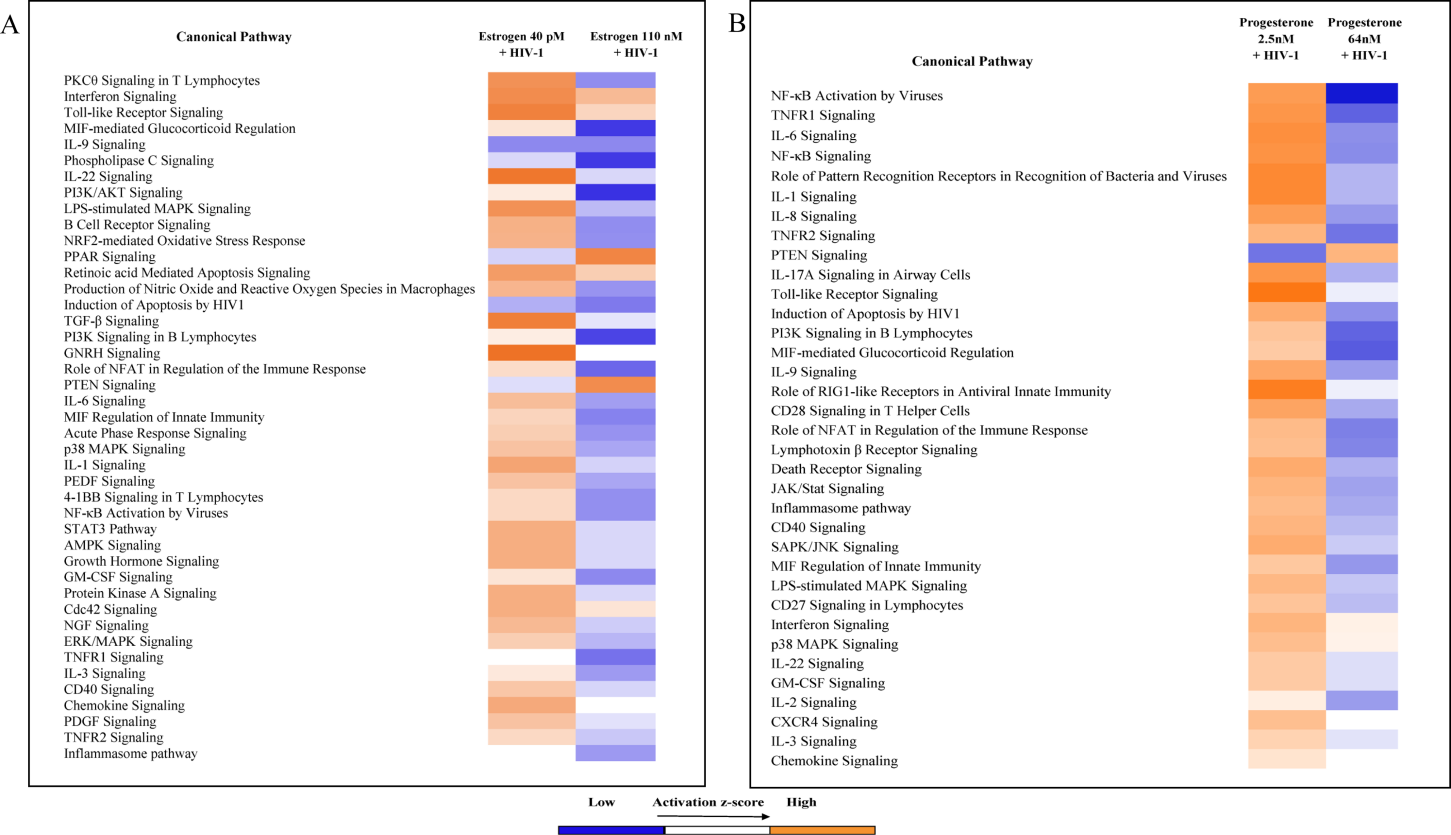
Ingenuity Pathway Analysis (IPA) of the differentially expressed host genes identified by the PCR array. Data sets were analyzed through the use of QIAGEN's Ingenuity® Pathway Analysis (IPA®, QIAGEN Redwood City, CA, USA www.qiagen.com/ingenuity). Identification of the canonical pathways from Ingenuity Pathways Knowledge Base (IPKB) most significantly associated with the genes differentially expressed between MDMs infected with HIV-1 pre-treated with estrogen (A) or progesterone (B) and MDMs infected with HIV-1 not treated with estrogen or progesterone samples. The differentially expressed genes identified by the PCR array were used for the analysis. The significance of the association was measured on the basis of the ratio of the number of genes from the data set that map to the pathway divided by the total number of genes that map to the canonical pathway (as displayed); and a p-value determining the probability that the association between the genes in the data set and the canonical pathway is explained by chance alone (Fischer’s exact test).

We used Ingenuity Pathway Analysis (IPA) to further explore the functional relationships between the differentially expressed gene sets identified by the PCR array for HIV-1 infected cells treated with 110 nM estrogen (high concentration) and 40 pM estrogen (low concentration) or 64 nM progesterone (high concentration) and 2.5 nM progesterone (low concentration). Comparison analysis identified a number of canonical pathways involved in PKC signaling, Interferon signaling, Toll-like receptor signaling, cytokine signaling and signal transduction pathways in cells treated with 40 pM or 110 nM estrogen. The differentially expressed genes in cells treated with 40 pM estrogen identified in these pathways exhibited a higher activation z-score compared to cells treated with 100 nM estrogen (Fig 5A), indicating that these genes were up regulated leading to the activation of these pathways. Similarly, (Fig 5B), canonical pathways comprising of genes involved in NFκB activation, TNFR1 signaling, Interferon, cytokine and chemokine signaling displayed significantly higher activation z-score in HIV-1 infected cell treated with 2.5 nM (low concentration) of progesterone compared to cells treated with 64 nM (high concentration of progesterone). Next, to understand the potential biological relevance of the differentially expressed genes in response to HIV-1 infection and hormone pre-treatment we used IPA Upstream Regulator Analysis and Diseases and Functions analysis (downstream effects). Upstream Regulator Analysis uses experimentally observed relationships between regulators and the gene expression data set to predict upstream transcriptional regulators. Diseases and Functions analysis predicts directional changes to the effected cellular processes or biological functions based on the gene expression data. Upstream Regulator analysis of the differentially expressed genes from HIV-1 infected cells pre-treated with low and high concentrations of estrogen and progesterone identified putative transcriptional regulators and other molecules that modulate gene expression. The results (S1 Table) indicate that in cells pre-treated with 40 pM estrogen, upstream regulators IL27, IRF7, TXN, and PI3K (family) were predicted to be activated and upstream regulators CD40LG and SMSRCA4 were predicted to be inhibited based on the activation z score. In cells pre-treated with 110 nM estrogen only SOX11 was predicted to be activated. In HIV-1 infected cells pre-treated with 2.5 nM progesterone several upstream regulators like transcription factors (NFKB1, RELA and P38 MAPK), Toll-like receptors (TLR2, TLR5, TLR6 and TLR7), Interferons (IFNG, IFNL1 and IFNA2), Interferon regulatory factors (IRF3 and IRF7), cytokines (IL27 and IL18) were predicted to be activated and upstream regulators like RNF216, IRF4, MAPK1, SOCS3, HMOX1, TNIP3, FASN, CD28 and CD3 were predicted to be inhibited based on the activation z score. In HIV-1 infected cells pre-treated with 64 nM progesterone upstream regulators IRF7 and CD28 were predicted to be activated and upstream regulators CD40LG, IL32, TIRAP, FceR1, IKBKB, PF4, ESCIT, CCL5, SP1 and SELPLG were predicted to be inhibited. Many Toll-like receptors, Interferons, Interferon regulatory factors and transcriptions factors identified as upstream regulator molecules by the analysis are potent regulators of cytokine and chemokine expression and their activation state indicates that pre-treatment with high concentrations of estrogen or progesterone promotes an anti-inflammatory environment.

Diseases and Functions analysis of the differentially expressed genes from infected cells pre-treated with 40 pM or 110 nM estrogen identified over 300 pathways that were significantly impacted (S2 Table). The results obtained from the Diseases and Functions analysis of the differentially expressed genes from infected cells pre-treated with 40 pM or 110 nM estrogen identified pathways involved inflammatory response, infectious disease and cell death that were predicted to have a decreased activation state. The results obtained from the analysis of the differentially expressed genes identified an increase in the predicted activation state in infected cells pre-treated with 2.5 nM and a decrease in the predicted activation state in cells treated with 64 nM progesterone of pathways involved in inflammatory response, gene expression and cell signaling (S2 Table). Thus, the results of the Diseases and Functions analysis also support the observation that pathways involved in inflammatory response, gene expression and cell signaling are down modulated in infected cells pre-treated with high concentrations of estrogen and progesterone leading to an anti-inflammatory environment.

## Discussion

The predominant influence of estrogen and progesterone in the transmission and replication of HIV may be due to their immunoregulatory functions [32]. Modulation of endogenous hormonal levels during the reproductive cycle exerts an influence on the secretion of antimicrobial peptides, the regulation of the functional properties of CD8+ cells, cytokine secretion and function, secretion and function of chemokines that attract inflammatory cells like neutrophils, macrophages and natural killer cells [33–35]. Fluctuations in endogenous hormonal levels are also known to alter immunological responses [36–38].

Epidemiological and challenge studies using the rhesus macaque model have described an association of increased risk of HIV-1 infection in humans and simian immunodeficiency virus (SIV) infection in macaques with the use of progesterone-based contraceptives. Furthermore, the use of progesterone-based contraceptives has been implicated in increased viral replication in the genital tract and acceleration disease progression [39]. Other studies have shown that subcutaneous implants of progesterone in non-human primates increased the transmission of SIV [40]. In addition, multiple reports have associated hormonal contraception with a higher rate of transmission; especially the usage of depomedroxyprogesterone acetate (DMPA) has been shown to have a 2- to 3-fold higher risk of HIV infection. A dose dependent correlation between progesterone levels and HIV-1 proliferation and increased shedding of HIV-1 in the genital tissue has been reported [36, 41]. Similarly, studies with non-human primates suggest that estrogen enhances the natural protective properties of the female genital tract tissue and decreases its susceptibility to virus transmission [42, 43]. Estrogen and its derivatives influence the migration and infiltration of lymphocytes, macrophages, NK cells and other cell types into the female genital tract. Estrogen also controls endometrial macrophage migration by inhibiting MCP-1 expression in endometrial stromal cells. High estrogen levels are known to down regulate ICAM-1, E-selectins and VCAM-1 leading to the decreased recruitment of inflammatory T cells and macrophages contributing to decreased susceptibility to HIV-1 transmission [36].

Others have reported that steroid hormones may exert an indirect regulatory effect on HIV-1 replication. These mechanisms include modulation of the synthesis of inflammatory cytokines or chemokines [44–46]. Studies have shown that a potent anti-inflammatory response is mediated by estrogen and progesterone treatment [16]. Reports in literature indicate that steroid hormones can exert a biphasic effect on cytokine secretion, low concentrations can up regulate secretion and high concentrations can down regulate secretion [47–50]. Consistent with our findings that high concentrations of estrogen down regulated expression of chemokines CXCL9, CXCL10/IP-10 and CXCL11 and pro-inflammatory cytokines IL-1β, IL-6, IL-18 and TNF-α and low concentrations of estrogen was associated with a slight increase in the secretion of IFN-a2, chemokines CXCL9 and CXCL11 and pro-inflammatory cytokines IL-6 and TNF-α (Fig 4A), others have shown that estradiol exerts a similar effect on the secretion of TNF-α; high concentrations of estrogen down regulate TNF-α secretion while low concentrations of estrogen up regulate TNF-α secretion [49, 51]. In addition, estrogen treatment at high concentrations attenuates the inflammatory response triggered by Tat protein, presumably by inhibiting NFκB interactions with promoter elements [52]. Progesterone treatment has also been associated with decreased expression of pro-inflammatory cytokines and chemokines MIP-1 α, MIP-1β and RANTES that are known to modulate HIV-1 infection [36, 53]. Data obtained in the present study indicates that pre-treatment with a high concentration of progesterone (64 nM) down regulated expression of chemokines CXCL9, CXCL10/IP-10 and CXCL11 and pro-inflammatory cytokines IL-1β, IL-6, IL-18 and TNF-α while treatment with low concentrations of progesterone increased the secretion of chemokines CXCL9, CXCL10/IP-10 and CXCL11 and pro-inflammatory cytokines IL-6 and TNF-α. Many studies have previously reported a correlation between the observed increase in the production of pro-inflammatory cytokines IL-1β, IL-6, IL-18 and TNF-α; and IFN inducible chemokines CXCL9, CXCL10 and CXCL11 during the course of HIV-1 infection and the up regulation of HIV-1 replication [19, 54–59]. Similarly other studies have reported observations correlating enhanced expression of pro-inflammatory factors and the stimulation of HIV-1 replication in ectocervival tissues from post-menopausal women with lower estrogen levels [60]. Taken together the slight increase in HIV-1 BaL replication observed in MDMs pre-treated with low concentrations of estrogen and progesterone could be due to the enhanced secretion of pro-inflammatory cytokines and chemokines. Consistent with these reports, our findings from the IPA analysis also indicate that pre-treatment with high concentrations of estrogen and progesterone promotes an anti-inflammatory environment and down modulates cytokine and chemokine production due to HIV-1 infection.

**Fig 6:**
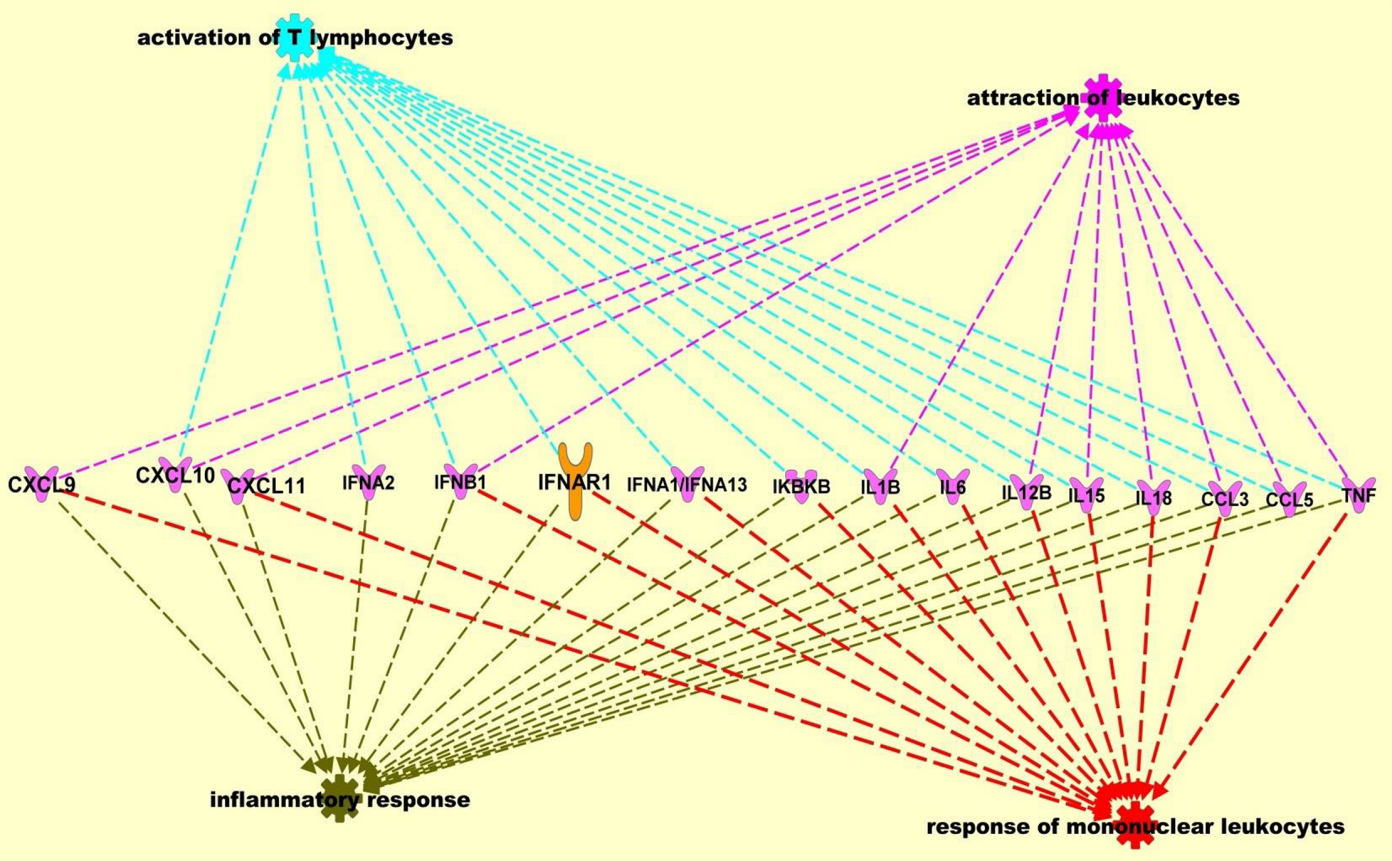
Ingenuity Pathway Analysis (IPA) of the differentially expressed host genes identified by the PCR array. Data sets were analyzed through the use of QIAGEN's Ingenuity® Pathway Analysis (IPA®, QIAGEN Redwood City, CA, USA www.qiagen.com/ingenuity). Network analysis included direct and indirect relationships with disease functions and selected genes differentially expressed between MDMs infected with HIV-1 pre-treated with estrogen or progesterone and MDMs infected with HIV-1 not treated with estrogen or progesterone samples.

Regulation of HIV-1 replication could also be mediated at the transcription level by estrogen and progesterone treatment. The HIV-1 LTR is known to contain hormone-response elements and the activation of hormone receptors by ligands could modulate HIV-1 transcription through direct interaction with these elements, or by regulating DNA binding and transcriptional activation of other cellular transcription factors [61]. Interactions of cellular transcription factors AP-1, SP-1, and NF-κB with estrogen receptor have been well documented [62]. Our results from the Upstream Regulator analysis also indicate that in HIV-1 infected cells, pre-treated with high concentrations of estrogen and progesterone, transcriptional regulators and other molecules that modulate expression of pro-inflammatory cytokine and chemokines were inhibited. This could lead to the down modulation of the pro-inflammatory cytokines and chemokines which in turn down regulate HIV-1 replication. Using IPA Pathway Designer tools to visualize pathways and networks of individual molecules, pro-inflammatory cytokine and chemokines modulated by hormone treatment we identified key pathways (Fig 6) involved in the inflammatory response, response of leukocytes, activation of lymphocytes and attraction of leukocytes.

## Conclusion

Our *in vitro* results indicate that high concentrations of estrogen and progesterone down regulated HIV-1 replication in MDMs, suggesting that these hormones exert a significant effect on HIV replication. In addition, our *in vitro* results suggest that treatment with high doses of estrogen and progesterone promotes an anti-inflammatory environment by down modulating the expression of pro-inflammatory cytokines and chemokines produced in response to HIV-1 infection. This down modulation of the early inflammatory response may facilitate inhibition of HIV-1 replication. Thus, the regulation of HIV-1 replication by steroid hormones may be a result of the interplay between transcriptional activation of the HIV-1 LTR and modulation of cytokine and chemokine expression and secretion. Further investigations will be necessary to determine the exact molecular mechanisms that contribute to the observed hormonal and sex specific effects and how they may affect clinical management of females vs. males in populations where diverse HIV subtypes and variants are prevalent. These findings may be relevant to clinical observations of sex specific differences in patient populations and point to the need for further investigation.

## Supporting information

S1 Table Upstream Regulator analysis of the differentially expressed host genes identified by the PCR array. (DOC)

S2 Table List of Diseases and Functions annotated by IPA functional analysis of the differentially expressed genes from infected cells pre-treated with 40 pM or 110 nM estrogen and 2.5 nM or 64 nM progesterone. (XLS)

S1 Fig Effect of Estrogen and progesterone treatment on CD4 receptor and CCR5 co-receptor expression in MDMs. Untreated and treated MDMs (1 x 10 ^6^) were harvested, fixed by incubation in 1% paraformaldehyde, washed twice with PBS, pH 7.4, and resuspended in FACS binding buffer (PBS, pH 7.4, containing 2% FBS, 0.1% NaN, 0.1% BSA, and 1 mg/ml human IgG). Cells were incubated with FITC-labeled monoclonal antibody to CD4, PE-labeled monoclonal antibody to CCR5 and control IgG (BD Biosciences Pharmingen, San Diego, CA) at 4°C for 30 min and washed three times with PBS, pH 7.4, fixed in 2% paraformaldehyde, and acquired for FACS analysis.
(TIFF)

S2 Fig Analysis of differentially expressed genes in HIV-1 infected MDMs not treated with estrogen or progesterone compared to control MDMs not treated with estrogen or progesterone and not infected with HIV-1 using RT^2^ Profiler PCR Arrays. RT^2^ Profiler PCR Array was used to examine the mRNA levels of different antiviral response genes in estrogen or progesterone treated MDMs infected with HIV-1 BaL and control MDMs not treated with estrogen or progesterone and not infected with HIV-1 BaL. Assays were performed with experimental RNA samples isolated from MDMs obtained from 3 independent donors.
(TIFF)
