# Supplementary material for: Modulation of HIV replication in monocyte derived macrophages (MDM) by steroid hormones
Source: PLoS One. 2018 Jan 26;13(1):e0191916. doi: 10.1371/journal.pone.0191916 (PMC5786332; doi:10.1371/journal.pone.0191916)
Supplement: S1 Table — (DOC) [file pone.0191916.s001.doc]

**Table 1: Upstream Regulator** Analysis of the differentially expressed host genes identified by the PCR array

| **Upstream Regulator** | **Hormone Treatment** | **Predicted Activation state** | **Activation z-score** | **p-value of overlap** |
| --- | --- | --- | --- | --- |
| **IL27** | 40 pM Estrogen | Activated | 2.224 | 1.29E-23 |
|  | 110 nM Estrogen |  | 0.349 | 1.29E-23 |
| **CD40LG** | 40 pM Estrogen |  | -0.282 | 3.37E-17 |
|  | 110 nM Estrogen | Inhibited | -2.572 | 3.37E-17 |
| **IRF7** | 40 pM Estrogen | Activated | 2.233 | 4.95E-12 |
|  | 110 nM Estrogen |  | 1.387 | 4.95E-12 |
| **TXN** | 40 pM Estrogen | Activated | 2 | 5.07E-10 |
|  | 110 nM Estrogen |  | 1 | 5.07E-10 |
| **PI3K (family)** | 40 pM Estrogen | Activated | 2.021 | 7.23E-10 |
|  | 110 nM Estrogen |  | 1.376 | 7.23E-10 |
| **Gsk3** | 40 pM Estrogen | Activated | 2.186 | 8.03E-08 |
|  | 110 nM Estrogen | Activated | 2.186 | 8.03E-08 |
| **SMARCA4** | 40 pM Estrogen |  | -0.557 | 1.65E-05 |
|  | 110 nM Estrogen | Inhibited | -2.414 | 1.65E-05 |
| **SOX11** | 40 pM Estrogen |  | 0 | 0.000236 |
|  | 110 nM Estrogen | Activated | 2 | 0.000236 |

| **Upstream Regulator** | **Hormone Treatment** | **Predicted Activation state** | **Activation z-score** | **p-value of overlap** |
| --- | --- | --- | --- | --- |
| **IFNG** | 2.5 nM Progesterone | Activated | 2.343 | 1.56E-34 |
|  | 64 nM Progesterone |  | 0.404 | 1.56E-34 |
| **IRF3** | 2.5 nM Progesterone | Activated | 2.495 | 6.76E-29 |
|  | 64 nM Progesterone |  | -0.552 | 6.76E-29 |
| **Interferon alpha** | 2.5 nM Progesterone | Activated | 3.489 | 3.35E-28 |
|  | 64 nM Progesterone |  | 0.988 | 3.35E-28 |
| **TLR7** | 2.5 nM Progesterone | Activated | 2.537 | 1.49E-26 |
|  | 64 nM Progesterone |  | -0.71 | 1.49E-26 |
| **TNF** | 2.5 nM Progesterone | Activated | 2.347 | 9.21E-24 |
|  | 64 nM Progesterone |  | -0.993 | 9.21E-24 |
| **IL27** | 2.5 nM Progesterone | Activated | 2.81 | 1.29E-23 |
|  | 64 nM Progesterone |  | 1.488 | 1.29E-23 |
| **P38 MAPK** | 2.5 nM Progesterone | Activated | 2.429 | 2.93E-23 |
|  | 64 nM Progesterone |  | 0.634 | 2.93E-23 |
| **NFKB (complex)** | 2.5 nM Progesterone | Activated | 2.006 | 5.52E-21 |
|  | 64 nM Progesterone |  | -1.069 | 5.52E-21 |
| **NFKB1** | 2.5 nM Progesterone | Activated | 2.565 | 1.01E-17 |
|  | 64 nM Progesterone |  | 0.298 | 1.01E-17 |
| **CD40LG** | 2.5 nM Progesterone |  | 1.043 | 3.37E-17 |
|  | 64 nM Progesterone | Inhibited | -2.048 | 3.37E-17 |
| **mir-146** | 2.5 nM Progesterone | Inhibited | -2.2 | 1.07E-16 |
|  | 64 nM Progesterone |  | 1.2 | 1.07E-16 |
| **IFNL1** | 2.5 nM Progesterone | Activated | 2.778 | 1.41E-15 |
|  | 64 nM Progesterone |  | 0.016 | 1.41E-15 |
| **TLR2** | 2.5 nM Progesterone | Activated | 2.092 | 2.08E-15 |
|  | 64 nM Progesterone |  | -1.911 | 2.08E-15 |
| **RELA**  **RELA** | 2.5 nM Progesterone | Activated | 2.432 | 2.14E-15 |
|  | 64 nM Progesterone |  | -0.561 | 2.14E-15 |
| **IFNA2** | 2.5 nM Progesterone | Activated | 2.607 | 7.05E-14 |
|  | 64 nM Progesterone |  | 0.259 | 7.05E-14 |
| **miR-146a-5p (and other miRNAs w/seed GAGAACU)** | 2.5 nM Progesterone | Inhibited | -2.345 | 2.08E-13 |
|  | 64 nM Progesterone |  | 1.066 | 2.08E-13 |
| **RNF216** | 2.5 nM Progesterone | Inhibited | -2.449 | 4.42E-13 |
|  | 64 nM Progesterone |  | 0 | 4.42E-13 |
| **IL32** | 2.5 nM Progesterone |  | 1.539 | 6.16E-13 |
|  | 64 nM Progesterone | Inhibited | -2.779 | 6.16E-13 |
| **MAPK1** | 2.5 nM Progesterone | Inhibited | -2.382 | 6.69E-13 |
|  | 64 nM Progesterone |  | -1.863 | 6.69E-13 |
| **IRF4** | 2.5 nM Progesterone | Inhibited | -2.63 | 1.26E-12 |
|  | 64 nM Progesterone |  | -0.478 | 1.26E-12 |
| **TIRAP** | 2.5 nM Progesterone |  | 1.247 | 1.31E-12 |
|  | 64 nM Progesterone | Inhibited | -2.225 | 1.31E-12 |
| **IRF7** | 2.5 nM Progesterone | Activated | 2.233 | 4.95E-12 |
|  | 64 nM Progesterone | Activated | 2.233 | 4.95E-12 |
| **FceR1** | 2.5 nM Progesterone | Activated | 2.027 | 4.95E-12 |
|  | 64 nM Progesterone | Inhibited | -2.57 | 4.95E-12 |
| **SOCS3** | 2.5 nM Progesterone | Inhibited | -2.213 | 1.55E-11 |
|  | 64 nM Progesterone |  | -0.391 | 1.55E-11 |
| **IL18** | 2.5 nM Progesterone | Activated | 2.326 | 1.36E-10 |
|  | 64 nM Progesterone |  | -0.345 | 1.36E-10 |
| **HMOX1** | 2.5 nM Progesterone | Inhibited | -2.567 | 2.94E-10 |
|  | 64 nM Progesterone |  | 0.263 | 2.94E-10 |
| **TNIP3** | 2.5 nM Progesterone | Inhibited | -2 | 3.19E-10 |
|  | 64 nM Progesterone |  | 1 | 3.19E-10 |
| **TLR5** | 2.5 nM Progesterone | Activated | 2.219 | 3.25E-10 |
|  | 64 nM Progesterone |  | -1.195 | 3.25E-10 |
| **IKBKB** | 2.5 nM Progesterone |  | 1.572 | 6.73E-10 |
|  | 64 nM Progesterone | Inhibited | -2.411 | 6.73E-10 |
| **TLR6** | 2.5 nM Progesterone | Activated | 2.219 | 1.01E-09 |
|  | 64 nM Progesterone |  | -1.387 | 1.01E-09 |
| **PF4** | 2.5 nM Progesterone |  | 1.498 | 1.01E-09 |
|  | 64 nM Progesterone | Inhibited | -2.2 | 1.01E-09 |
| **ECSIT** | 2.5 nM Progesterone |  | 1.342 | 1.14E-09 |
|  | 64 nM Progesterone | Inhibited | -2.39 | 1.14E-09 |
| **miR-155-5p (miRNAs w/seed UAAUGCU)** | 2.5 nM Progesterone |  | -1.404 | 1.86E-09 |
|  | 64 nM Progesterone | Activated | 2.406 | 1.86E-09 |
| **FASN** | 2.5 nM Progesterone | Inhibited | -2 | 1.11E-08 |
|  | 64 nM Progesterone |  | 1 | 1.11E-08 |
| **CCL5** | 2.5 nM Progesterone |  | 0.816 | 2.12E-08 |
|  | 64 nM Progesterone | Inhibited | -2.449 | 2.12E-08 |
| **LILRA2** | 2.5 nM Progesterone | Activated | 2 | 6.57E-08 |
|  | 64 nM Progesterone |  | -1 | 6.57E-08 |
| **TP53** | 2.5 nM Progesterone | Activated | 2.434 | 8.89E-08 |
|  | 64 nM Progesterone |  | -1.927 | 8.89E-08 |
| **SP1** | 2.5 nM Progesterone |  | 0.97 | 0.000000117 |
|  | 64 nM Progesterone | Inhibited | -2.425 | 0.000000117 |
| **SELPLG** | 2.5 nM Progesterone |  | 0.447 | 0.000000144 |
|  | 64 nM Progesterone | Inhibited | -2.236 | 0.000000144 |
| **CD28** | 2.5 nM Progesterone | Inhibited | -2.363 | 0.00000143 |
|  | 64 nM Progesterone | Activated | 2.363 | 0.00000143 |
| **CD3** | 2.5 nM Progesterone | Inhibited | -2.187 | 0.0000716 |
|  | 64 nM Progesterone |  | 1.522 | 0.0000716 |
