# Supplementary figures and images for: Modulation of HIV replication in monocyte derived macrophages (MDM) by steroid hormones
Source: PLoS One. 2018 Jan 26;13(1):e0191916. doi: 10.1371/journal.pone.0191916 (PMC5786332; doi:10.1371/journal.pone.0191916)

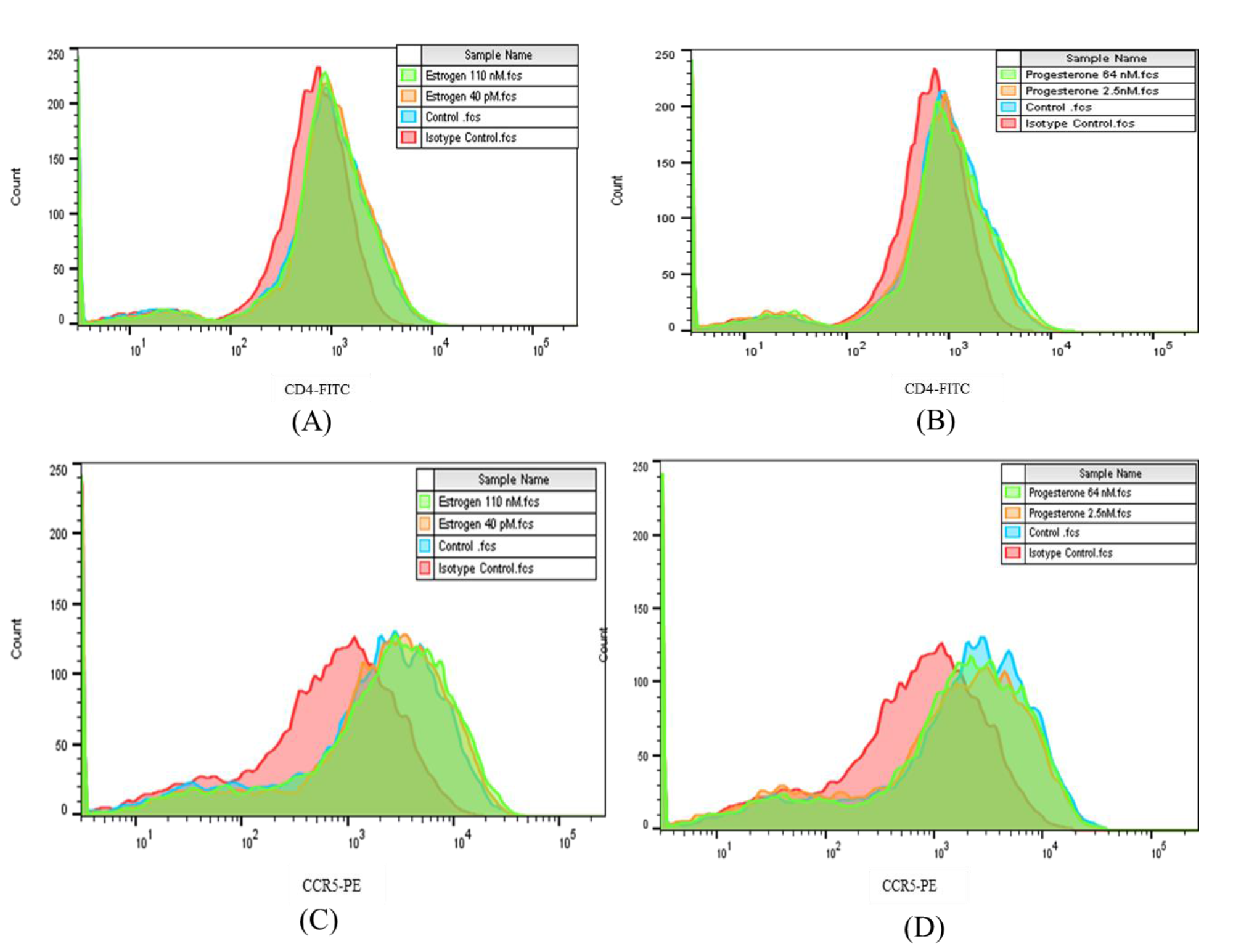

Supplement: S1 Fig — Untreated and treated MDMs (1 x 10 6) were harvested, fixed by incubation in 1% paraformaldehyde, washed twice with PBS, pH 7.4, and resuspended in FACS binding buffer (PBS, pH 7.4, containing 2% FBS, 0.1% NaN, 0.1% BSA, and 1 mg/ml human IgG). Cells were incubated with FITC-labeled monoclonal antibody to CD4, PE-labeled monoclonal antibody to CCR5 and control IgG (BD Biosciences Pharmingen, San Diego, CA) at 4°C for 30 min and washed three times with PBS, pH 7.4, fixed in 2% paraformaldehyde, and acquired for FACS analysis. (TIFF) [file pone.0191916.s003.tiff]

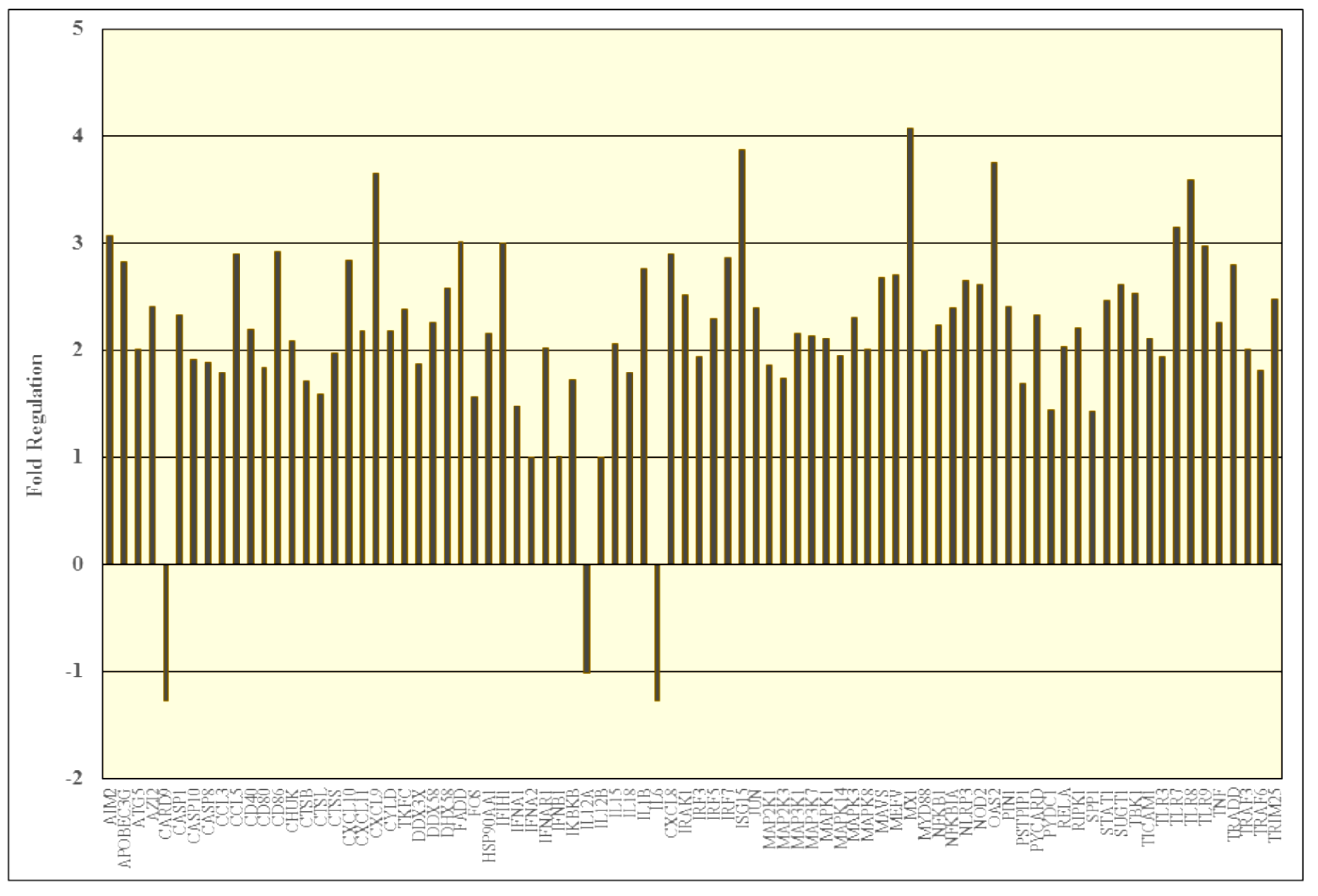

Supplement: S2 Fig — RT2 Profiler PCR Array was used to examine the mRNA levels of different antiviral response genes in estrogen or progesterone treated MDMs infected with HIV-1 BaL and control MDMs not treated with estrogen or progesterone and not infected with HIV-1 BaL. Assays were performed with experimental RNA samples isolated from MDMs obtained from 3 independent donors. (TIFF) [file pone.0191916.s004.tiff]
